# Insight Into Chromatin-Enriched RNA: A Key Chromatin Regulator in Tumors

**DOI:** 10.3389/fcell.2021.649605

**Published:** 2021-04-14

**Authors:** Jixing Zhang, Tianyi Ding, He Zhang

**Affiliations:** ^1^Institute for Regenerative Medicine, Shanghai East Hospital, School of Life Science and Technology, Tongji University, Shanghai, China; ^2^Frontier Science Research Center for Stem Cells, Tongji University, Shanghai, China

**Keywords:** chromatin-enriched RNA, tumor, cancer stem cell, chromatin loop, R-loop

## Abstract

Chromatin-enriched RNAs (cheRNAs) constitute a special class of long noncoding RNAs (lncRNAs) that are enriched around chromatin and function to activate neighboring or distal gene transcription. Recent studies have shown that cheRNAs affect chromatin structure and gene expression by recruiting chromatin modifiers or acting as bridges between distal enhancers and promoters. The abnormal transcription of cheRNAs plays an important role in the occurrence of many diseases, particularly tumors. The critical effect of cancer stem cells (CSCs) on the formation and development of tumors is well known, but the function of cheRNAs in tumorigenesis, especially in CSC proliferation and stemness maintenance, is not yet fully understood. This review focuses on the mechanisms of cheRNAs in epigenetic regulation and chromatin conformation and discusses the way cheRNAs function in CSCs to deepen the understanding of tumorigenesis and provide novel insight to advance tumor-targeting therapy.

## Introduction

As one of the main causes of death worldwide, cancer impedes the increase in people’s life expectancy. It is estimated that approximately 9.6 million people died of cancer in 2018 (Bray et al., 2018). The reasons of tumor formation are complex and involve many factors, which also increases the difficulty of tumor therapy. Studies have shown that somatic copy-number changes (Beroukhim et al., 2010), epigenetic changes (Kundaje et al., 2015), and mutations in noncoding regions (Melton et al., 2015) are deeply associated with tumorigenesis. Compared to protein-coding genes, those noncoding genes account for more than 98% of the entire human genome, in which mutations may lead to tumorigenesis. Genome-wide association analysis (GWAS) indicates that noncoding genome mutations are relevant to a variety of diseases, including tumors (Maurano et al., 2012).

According to transcriptome analysis, human cells generate a large number of transcripts, of which over 68% were classified as long noncoding RNAs (lncRNAs; Iyer et al., 2015). LncRNAs constitute a class of functional RNA molecules with a length of more than 200 nucleotides and lacking protein coding ability. They are usually located in the nucleus and enriched in chromatin and specific subnuclear compartments (Sun et al., 2018). As a unique subgroup of lncRNAs, chromatin-enriched RNAs (cheRNAs) that are generally transcribed by RNA polymerase II and enriched around chromatin, and can transcriptionally activate neighboring genes (Werner and Ruthenburg, 2015). CheRNA can be regarded as a new kind of enhancer RNA, but their strand length, histone modification markers, and strand-specificity are different (Gayen and Kalantry, 2017). CheRNAs are lineage specific, which can stimulate gene transcription in *cis* by promoting contacts to a downstream enhancer (Werner et al., 2017). In addition, cheRNAs function at multiple chromatin levels, including chromatin modification (Khalil et al., 2009), chromosome inactivation (Brown et al., 1992), nuclear structure changes (Bergmann and Spector, 2014; Quinodoz and Guttman, 2014; Engreitz et al., 2016), and transcription (Ponting et al., 2009; Vance and Ponting, 2014). Recent studies have shown that transcription disorders associated with cheRNAs are deeply relevant to the progression of a number of tumors (Gupta et al., 2010; Martens-Uzunova et al., 2014; Huarte, 2015; Schmitt and Chang, 2016). Considering the importance of cheRNA regulation at different chromatin levels, it is necessary to study its effects on tumorigenesis.

Tumors comprise a complicated group of heterogeneous cells, other than a collection of a single type of cloned tumor cell. Due to the influence of heredity, epigenetics, and the complex tumor microenvironment, tumor cells show a high degree of heterogeneity (Magee et al., 2012; Nguyen et al., 2012; Meacham and Morrison, 2013). Tumor heterogeneity is the major factor characterizing tumor metastasis and recurrence, and it is also a key barrier to tumor therapy (Meacham and Morrison, 2013; Saygin et al., 2019). Hence, it is assumed that certain types of cells in tumors are responsible for maintaining tumor growth and metastasis. Generally, the cells presenting the greatest tumor propagating potential could be regarded as the cancer stem cells (CSCs; Greaves and Maley, 2012; Beck and Blanpain, 2013). CSCs hold the properties of stem cells, such as self-renewal capacity, tumor differentiation potential, and stem gene expression (Kreso and Dick, 2014; Batlle and Clevers, 2017; Suva and Tirosh, 2020). The CSC model provides an explanation suggesting that a small number of CSCs can maintain the heterogeneity and occurrence of tumors and differentiate into cells with distinct hierarchical structures (Reya et al., 2001; Shackleton et al., 2009; Kreso and Dick, 2014). In 1994, John Dick et al. provided a model for CSC research in acute myeloid leukemia (AML; Lapidot et al., 1994; Bonnet and Dick, 1997). To date, CSCs have been identified in a variety of tumor tissues, including breast cancer (Al-Hajj et al., 2003), colon cancer (Ricci-Vitiani et al., 2007), brain tumors (Singh et al., 2004), pancreatic cancer (Hermann et al., 2007), melanoma (Schatton et al., 2008), gastric cancer (Takaishi et al., 2008), and liver cancer (Sell and Leffert, 2008). Previous studies have shown that CSCs play significant roles in tumor growth and migration (Peitzsch et al., 2017). Shimokawa et al. (2017) found that Lgr5+ colon CSCs had the potential for self-renewal and differentiation, driving the growth of colon cancer, and tumorigenesis can be regressed once the CSCs are lost. Pancreatic cancer is a malignant tumor with a high fatality rate, partly due to its persistent metastasis, in which pancreatic CSCs play crucial roles. Furthermore, the CD133+ and CXCR4+ pancreatic CSCs control the metastasis of pancreatic cancer, and their ablation can lead to the loss of the pancreatic cancer metastatic phenotype (Hermann et al., 2007). This review describes chromatin-related cheRNAs as the anchor point, trying to clarify the cheRNA-induced imbalance in CSCs and tumors at the chromatin level, thereby providing a new perspective for understanding tumorigenesis and tumor-targeting therapy.

## The Role of CheRNAs in Nucleosome Positioning

**Figure 1:**
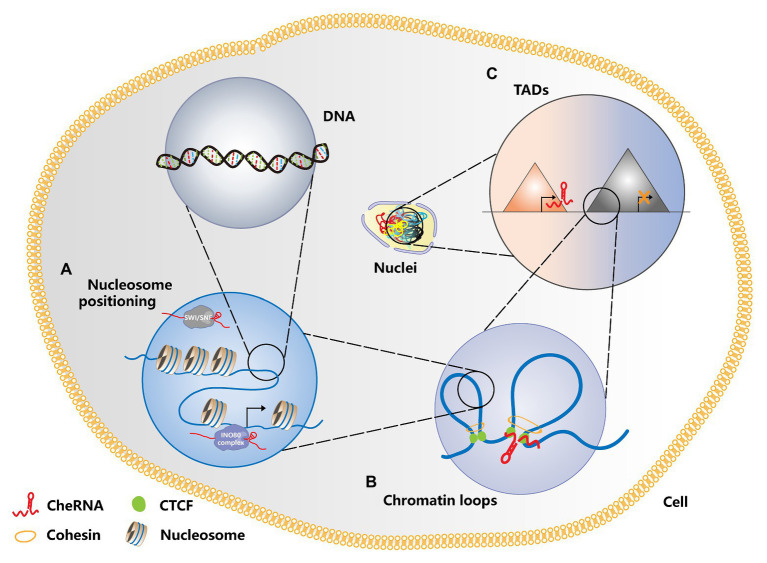
Schematic diagram of cheRNAs regulating chromosome conformation. **(A)** CheRNAs antagonize the SWI/SNF complex and prevent its binding to nucleosomes, thereby changing the nucleosome positioning and regulating gene transcription. In contrast, cheRNA recruits the INO80 complex, disaggregates the nucleosomes in the gene promoter region, and activates gene transcription. **(B)** CheRNAs can participate in the formation of chromatin loops in the promoter and enhancer regions, activate gene expression, and promote tumorigenesis. **(C)** Whether cheRNAs are transcribed or not can change the topologically associating domain (TAD) conformation of relevant regions, thereby regulating gene expression patterns and affecting tumor progression. TADS are represented by different triangles. The red one and the gray one, respectively shows the TADs where cheRNAs are transcribed or not transcribed.

Nucleosome remodeling is a key factor in gene expression, and chromatin remodeling agents are involved in this process (Segal and Widom, 2009). ATP-dependent chromatin remodelers can be divided into four main families: switch/sucrose non-fermentable (SWI/SNF), ISWI, CHD, and INO80 (Portela and Esteller, 2010; You and Jones, 2012). A growing number of studies have clarified that chromatin remodeling agents are deeply connected with the occurrence of tumors (Clapier and Cairns, 2009; Ho and Crabtree, 2010; Hargreaves and Crabtree, 2011; Wilson and Roberts, 2011). CheRNA-mediated nucleosome localization has been reported in the *Arabidopsis*, yeast, and animals, indicating that this biological process has universal significance (Marina et al., 2013; Zhu et al., 2013; Zhao et al., 2016). Similarly, cheRNA-mediated nucleosome localization also plays a critical role in tumorigenesis. The cheRNA *SChLAP1* is highly expressed in prostate cancer, which promotes the invasion and metastasis of prostate cancer cells by antagonizing the SWI/SNF complex and then affecting the localization of nucleosomes (Prensner et al., 2013). In view of the key roles of CSCs in tumorigenesis and metastasis, it is significant to understand their own regulatory mechanisms. Therefore, we emphasize the essential part of cheRNA in CSC proliferation and stemness maintenance. The cheRNA *HAND2-AS1* is highly expressed in liver stem cells. By recruiting the INO80 chromatin remodeling complex to the promoter region of *BMPR1A*, *HAND2-AS1* maintains the self-renewal of liver stem cells and promotes the occurrence of liver cancer (Wang et al., 2019b). Besides, the cheRNA *lncTCF7* recruits the chromatin remodeling complex SWI/SNF to the promoter region of *TCF7* to activate the Wnt signaling pathway, thereby promoting the self-renewal of liver CSCs (Wang et al., 2015). As a member of the CHD family, the Mi-2/NuRD complex plays a critical role in genome stability and tumor biology (Clapier and Cairns, 2009; Lai and Wade, 2011; Xia et al., 2017). The Mi-2/NuRD complex is recruited by cheRNA *LncHDAC2* to repress *PTCH1* transcription, which contributes to enhancing the Hedgehog signaling pathway and advancing the self-renewal of liver CSCs (Wu et al., 2019b). Figure 1A depicts a schematic diagram of cheRNAs regulating nucleosome positioning. Currently, cheRNAs are known to mainly regulate the conformation of different chromatin regions by recruiting or rejecting chromatin remodeling complexes, thus further changing gene expression. However, it remains unclear whether other RNA or protein factors are involved in this process, and therefore, the potential roles of these factors need to be further explored.

## CheRNAs Mediate Chromatin Loops

Numerous researches have shown that chromatin conformation is closely correlated with gene expression regulation, especially the long-distance ones (Sexton and Cavalli, 2015; Uhler and Shivashankar, 2017). The most common mode of long-distance chromatin interaction is that of promoters and distal enhancers (Li et al., 2012; Sanyal et al., 2012). These chromatin-loop-mediated promoter and enhancer interactions also hold essential roles in the formation of induced pluripotent stem cells (Zhang et al., 2013). In addition to the most common proteins, such as CTCF, the SMC complex, and Cohesin, recent studies have found that cheRNAs can also modulate the chromatin loop formation of an active enhancer region and its target promoter (Orom and Shiekhattar, 2013; Yang et al., 2013; Trimarchi et al., 2014). The highly expressed cheRNAs *PRNCR1* (also known as *PCAT8*) and *PCGEM1* in prostate cancer can bind to the androgen receptor and mediate the cyclization of the promoters of androgen receptor binding enhancers and their target genes to promote the development of tumors (Yang et al., 2013). In T-cell acute lymphoblastic leukemia (T-ALL), the Notch-regulated cheRNA *LUNAR1* binds to the enhancer of *IGF1R*, activates the promoter of *IGF1R* in *cis*, maintains a high level of mRNA expression, and promotes the growth of T-ALL (Trimarchi et al., 2014). The function of cheRNA in the formation of chromatin loops is explained in Figure 1B. These studies indicated that cheRNAs promote the circularization of enhancer and promoter interactions in different regions by recruiting chromatin regulators, thereby affecting the expression of downstream genes. However, it is still unclear that how cheRNAs participate in the formation of chromatin loops, and further in-depth studies are needed to determine the mechanism.

## CheRNA Regulation of Topologically Associating Domains

Previously, the eukaryotic genome was generally regarded as a one-dimensional entity composed of linear DNA sequences. However, with the development of chromatin conformation capture technology, people have gradually realized that chromatin constitutes a complex, ordered three-dimensional spatial structure. The double helix of DNA and histone octamers is packaged into nucleosomes, which are further folded to form chromatin fibers (Bonev and Cavalli, 2016). Chromatin fibers form rings, and multiple chromatin rings are assembled to form chromosome domains or topologically associating domains (TADs), which are megabases (Dixon et al., 2012; Nora et al., 2012). The interaction between multiple TADs leads to the formation of chromosomal compartments, and chromatin compartments further form chromatin territories (Bonev and Cavalli, 2016). A large number of studies have shown that TADs have major roles in gene expression (Andrey et al., 2013), disease occurrence (Kloetgen et al., 2020), and mammalian embryonic development (Ke et al., 2017). TAD disorders are deeply relevant to tumorigenesis. The depletion of CTCF and Cohesin weakens the formation of TADs, indicating that they can participate in the formation of the TAD boundary (Phillips-Cremins et al., 2013; Wutz et al., 2017). Studies have revealed that cheRNAs can participate in changes in the TAD boundary during immunoglobulin class transition (Rothschild et al., 2020). In the same manner, there are relevant studies in tumors. The cheRNA *HOTTIP* was upregulated in AML, and its knockout inhibited the expression of genes related to leukogenesis. Mechanistically, *HOTTIP* can alter HOXA-driven TAD and transcription program, leading to the occurrence of AML (Luo et al., 2019). In addition, *HOTTIP* modulates the stemness maintenance of pancreatic CSCs by enhancing the Wnt/β-catenin pathway (Fu et al., 2017). Patients with high *HOTTIP* expression had shorter survival than those with low expression. Furthermore, the high expression of *HOTTIP* is correlated with lymph node metastasis, indicating that *HOTTIP* may be a biomarker for lymph node metastasis in patients with pancreatic cancer and provide a basis for pancreatic cancer targeting therapy. Figure 1C shows whether transcription of cheRNA can alter TAD conformation. Changes in TAD boundaries are very important for gene expression regulation. Previous studies have indicated that CTCF occupies most of TAD boundaries and usually function with cohesion complex. However, little is known about the involvement of cheRNAs in the regulation of the TAD boundary, which should be paid more attention to so that we can discover new models of gene expression regulation. Table 1 summarizes the regulatory role of RNA in chromatin conformation in tumors and CSCs.

**Table 1:** Summary of chromatin-enriched RNAs (cheRNAs) modulate chromosome conformation in cancers and cancer stem cells (CSCs).

	CheRNA type	Expression	Potential target(s)	Cancer or CSC types	References
Nucleosome positioning	SChLAP1	Upregulated	SWI/SNF	Prostate cancer	Prensner et al., 2013
	HAND2-AS1	Upregulated	BMPR1A	Hepatocellular carcinoma	Wang et al., 2019b
	lncTCF7	Upregulated	TCF7	Liver cancer stem cell	Wang et al., 2015
	LncHDAC2	Upregulated	NuRD complex	Liver cancer stem cell	Wu et al., 2019b
Chromatin loop	PRNCR1	Upregulated	Androgen receptor	Prostate cancer	Yang et al., 2013
	PCGEM1	Upregulated	Androgen receptor	Prostate cancer	Yang et al., 2013
	LUNAR1	Upregulated	IGF1R	T cell acute lymphoblastic leukemia	Trimarchi et al., 2014
TAD	HOTTIP	Upregulated	HOXA	Acute myeloid leukemia	Luo et al., 2019

## CheRNAs Guide DNA and Histone Modification

Epigenetic modification is the covalent modification of DNA, histones, or nucleosomes that does not affect the coding sequence of the genome, but can change the expression of genes (Egger et al., 2004; Allis and Jenuwein, 2016). The dysregulation of the epigenetic regulatory network can lead to inappropriate transcription or silencing of certain genes, thereby inducing tumorigenesis (Jones and Baylin, 2002; Egger et al., 2004). CheRNA-mediated epigenetic modification is involved in the development of a variety of cancers.

**Figure 2:**
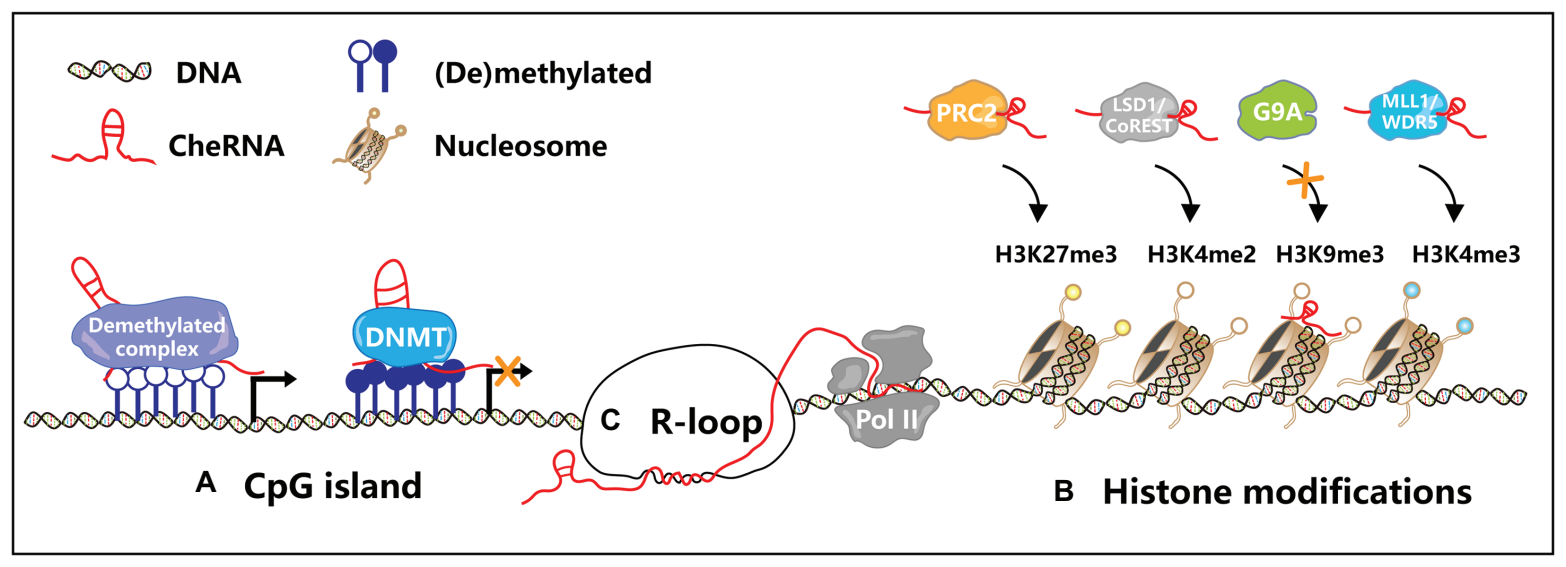
Transcription regulation by cheRNAs. **(A)** In the CpG island region, cheRNAs recruit DNA methyltransferase (DNMT) or demethylated complex to regulate gene transcription. **(B)** CheRNAs recruit PRC2 and MLL1/WDR5 complexes, mediate specific regions H3K27me3 and H3K4me3, and then inhibit or activate gene expression. Similarly, cheRNAs recruit the LSD1/CoREST complex and promote H3K4me2 demethylation. On the contrary, cheRNAs repel G9A, inhibit H3K9 trimethylation, and activate gene expression. **(C)** The antisense cheRNAs form R-loops in the gene promoter region, maintain the open state of chromatin, and promote the gene transcription.

The hypomethylation status of the tumor genome was one of the first epigenetic factors to be studied (Feinberg and Vogelstein, 1983). DNA methylation is usually regarded as a mark of epigenetic silencing, and its dysregulation is deeply related to the occurrence of many tumors (Egger et al., 2004; Jones and Baylin, 2007; Chin et al., 2008; Muzny et al., 2012). DNA methylation prevents other DNA-binding proteins from binding to the genome at a specific site, leading to transcriptional silencing. In recent years, studies have illustrated that cheRNAs can also participate in DNA methylation modification, which in turn affects gene expression. The cheRNA *LUCAT1* promotes the proliferation and metastasis of esophageal squamous cell carcinoma (ESCC) by increasing the stability of DNA methyltransferase 1 (DNMT1) and increases the methylation level of tumor suppressor factors, leading to the proliferation and metastasis of ESCC (Yoon et al., 2018). In colon cancer, the expression of cheRNA *DACOR1* is inhibited, and induction of *DACOR1* leads to the remethylation of CpG islands at *FOS* and *JUN* promoter sites, thereby affecting the development of colon cancer (Somasundaram et al., 2018). In addition, the cheRNA can also participate in DNA demethylation. CheRNA *TARID* recruits the DNA demethylation regulator GADD45A to the promoter region of *TCF21* and mediates its demethylation to activate the expression of the tumor suppressor TCF21 (Arab et al., 2014). Furthermore, in CSCs, cheRNA can also participate in the formation of DNA methylation. CheRNA *DLX6-AS1* downregulates the expression of the tumor suppressor CADM1 by increasing the methylation level of its promoter region, thus activating the STAT3 signaling pathway to promote the self-renewal of liver CSCs (Wu et al., 2019a). Figure 2A illustrates how cheRNAs regulate the DNA methylation level of the promoter region to affect gene transcription. Besides, other studies have shown that the occupation of cheRNA in the promoter region can suppress gene expression and regulate the progression of CSC. By competitively binding IL-6 promoter with NF-κB, *lnc-DILC* inhibits the autocrine IL-6/STAT3 signaling, therefore suppressing liver CSC expansion (Wang et al., 2016b). Clinical analysis shows that generally hepatocellular carcinoma patients have lower levels of *lnc-DILC*, while some patients with higher levels of *lnc-DILC* have lower recurrence rate and longer survival time. This research indicates that *lnc-DILC* may be a potential prognostic marker of hepatocellular carcinoma, and it can be utilized as a potential therapeutic target for liver CSCs.

Abnormal histone modification patterns are involved in many aspects of tumorigenesis. The cheRNA *HOTAIR* acts as a scaffold for the histone modification complexes PRC2 and LSD1/CoREST/REST, thereby coupling histone H3K27 methylation and H3K4 demethylation (Tsai et al., 2010). The abnormal transcription of cheRNA *ROR* blocks the binding of histone methyltransferase G9A to the oncogene *TESC* promoter region, and thus promotes *TESC* expression by inhibiting histone H3K9 methylation, leading to tumorigenesis (Fan et al., 2015). The cheRNA *ZEB1-AS1* was shown to promote the occurrence of prostate cancer *via* recruiting histone methyltransferase MLL1 to the promoter region of ZEB1, inducing H3K4me3 modification, and thus activating ZEB1 transcription (Su et al., 2017). Moreover, the cheRNA *GAS8-AS1* forms a complex with MLL1 and WDR5 to facilitate the formation of H3K4me3 in the GAS8 promoter region, thus enhancing the activity of RNA polymerase II, promoting the expression of GAS8, and accelerating the occurrence of liver cancer (Pan et al., 2018). *In vitro* and *in vivo* experiments have shown that inhibition of cheRNA *00511* represses cell proliferation and migration, whereas cheRNA *00511* upregulation enhances tumorigenesis *via* serving as a scaffold for the EZH2/PRC2 complex to target p57 H3K27 methylation in NSCLC cell lines (Sun et al., 2016a). Gastric cancer-related lncRNA 1 (*GClnc1*) is upregulated in gastric cancer and is associated with gastric cancer occurrence, metastasis, and poor prognosis. *GClnc1* recruits WDR5 (a histone methyltransferase) to the promoter region of the superoxide dismutase 2 mitochondrial (SOD2, a mitochondrial enzymes) and KAT2A (a histone acetyltransferase), which in turn promotes H3K4 trimethylation and H3K9 acetylation levels, thereby promoting the expression of SOD2 (Sun et al., 2016b). High expression of cheRNA *GClnc1* leads to lager tumor volume, higher vascular infiltration, and lower survival rate of patients, indicating that it can be regarded as a biomarker for gastric cancer. Uveal melanoma (UM) is one of the most common malignant tumors in the eyes of adults. Studies have shown that cheRNA *CANT1* can bind to the promoter regions of *JPX* and *FTX* and further increase the expression of the H3K4me3 to impede UM tumorigenesis (Xing et al., 2017). Conversely, cheRNA *PAUPAR* modulates *HES1* transcription *via* repressing H3K4me3, which significantly reduces UM growth and metastasis (Ding et al., 2016).

In colon CSCs, the cheRNA *Lnc34a* simultaneously recruits HDAC1 and Dnmt3a, modulates histone deacetylation and DNA methylation in the miR-34a promoter region, suppresses Lnc34a expression, and promotes colon CSC proliferation and self-renewal (Wang et al., 2016a). Furthermore, inhibition of cheRNA *HotairM* modulates *HOXA1* epigenetic silencing *via* histone H3K27 trimethylation, which promotes the self-renewal of colorectal CSCs through the HOXA1-Nanog signal loop (Li et al., 2020). About cheRNAs modulate histone modification to alter gene transcription is depicted in Figure 2B. It has been reported that cheRNAs induce epigenetic modification in numerous tumors and CSCs, indicating that this biological process is of great significance for tumorigenesis.

## CheRNAs are Involved the Formation of R-Loops

The R-loop consists of the hybrid chain formed by RNA and DNA and a replaced single-stranded DNA, which is generally produced during DNA replication or transcription (Santos-Pereira and Aguilera, 2015). Previously, people usually believed that the R-loop was a byproduct of transcription, while recent studies have shown that the R-loop plays a vital role in gene expression and genome stability. Generally, R-loops can be categorized as a physiological R-loop or a pathological R-loop. Physiological R loops occur in normal biological processes, such as B cell immunoglobulin class switching (Yu et al., 2003), mitochondrial DNA replication, or *Escherichia coli* ColE1 plasmid replication (Aguilera and Garcia-Muse, 2012). The excessive accumulation of R-loops and pathological R-loops affects DNA replication, transcription, and genome stability (Aguilera and Garcia-Muse, 2012; Santos-Pereira and Aguilera, 2015; Sollier and Cimprich, 2015; Garcia-Muse and Aguilera, 2019). The formation of the R-loop is closely associated with the occurrence of a variety of human diseases and may be responsible for the imbalance of chromatin homeostasis in tumors (Bhatia et al., 2014; Santos-Pereira and Aguilera, 2015).

The recent finding is that the production of R-loops is deeply relevant to cheRNAs, especially antisense cheRNAs. CheRNA-mediated R-loop formation has been reported in *Arabidopsis thaliana* (Sun et al., 2013), yeast (Cloutier et al., 2016), and mammalian cells (Boque-Sastre et al., 2015). Similarly, cheRNA-mediated R-loop formation also contributes to tumorigenesis (Figure 2C). In colon cancer, the antisense cheRNA *VIM-AS1* forms an R-loop near the VIM transcription start site, which upregulates the expression of VIM by maintaining the open state of chromatin (Boque-Sastre et al., 2015). Besides, the antisense cheRNA *TARID* forms an R-loop in the promoter region of the tumor suppressor TCF21. GADD45A binds to the R-loop and recruits the DNA demethylase TET1 to modulate the expression of TCF21 (Arab et al., 2019). Although cheRNAs are involved in the formation of R-loops in tumor cells, how cheRNA-mediated R-loops function in CSCs remains unclear. Further exploration of the function and mechanism of R-loops induced by cheRNAs in CSCs is needed, which might inspire novel insights into tumor therapy. The role of cheRNAs in transcription regulation is generalized in Table 2.

**Table 2:** The role of cheRNAs in transcription regulation in cancers or CSCs.

	CheRNA type	Expression	Potential target(s)	Cancer or CSC types	References
DNA methylation and demethylation	LUCAT1	Upregulated	DNMT1	Esophageal squamous cell carcinoma (ESCC)	Yoon et al., 2018
	DACOR1	Downregulated	ATF	Colon cancer	Somasundaram et al., 2018
	TARID	Downregulated	TCF21	Non-small cell lung cancer, head and neck squamous cell carcinomas, and ovarian cancers	Arab et al., 2014
	DLX6-AS1	Upregulated	CADM1	Liver cancer stem cell	Wu et al., 2019a
	lnc-DILC	Downregulated	IL-6	Liver cancer stem cell	Wang et al., 2016b
Histone modification	HOTAIR	Downregulated	PRC2/LSD1	HeLa	Tsai et al., 2010
	ROR	Upregulated	TESC	Gastric cancer and colon cancer	Fan et al., 2015
	ZEB1-AS1	Upregulated	ZEB1	Prostate cancer	Su et al., 2017
	GAS8-AS1	Downregulated	GAS8	Hepatocellular carcinoma	Pan et al., 2018
	LINC00511	Upregulated	p57	Non-small-cell lung cancer	Sun et al., 2016a
	GClnc1	Upregulated	SOD2	Gastric cancer	Sun et al., 2016b
	CANT1	Downregulated	JPX/FTX/XIST	Uveal melanoma	Xing et al., 2017
	PAUPAR	Downregulated	HES1	Uveal melanoma	Ding et al., 2016
	Lnc34a	Upregulated	miR-34a	Colon cancer stem cells	Wang et al., 2016a
	HotairM1	Downregulated	HOXA1	Colorectal carcinoma stem cell	Li et al., 2020
R-LOOP	VIM-AS1	Downregulated	VIM	Colon cancer	Boque-Sastre et al., 2015
	TARID	Downregulated	TCF21	Head and neck squamous cell carcinomas	Arab et al., 2019

## Discussion

The mechanism of tumorigenesis is complicated, and the imbalance of chromatin homeostasis plays a crucial role in it (Yu and Ren, 2017). Our previous researches showed that the aberrant open chromatin status at chr12p13.3 induces a novel cheRNA *GAU1* and *cis* activates the expression of oncogene *GALNT8* by recruiting the transcription elongation factor TCEA1, which accelerates tumor progression (Chai et al., 2018). On the contrary, cheRNAs can also affect chromatin homeostasis in many ways, such as nucleosome localization, TAD conformation, and R-loop formation. With the application of transcriptomics and computational biology, more and more cheRNAs are found in tumors and CSCs. Numerous studies have shown that cheRNAs function in tumor growth, metastasis, drug resistance, angiogenesis, and CSC stemness maintenance (Gutschner and Diederichs, 2012; Xiong et al., 2016). In summary, more researches have drawn attention to the role of cheRNAs participating chromatin regulation in tumors and CSCs.

With high tissue and cell specificity, cheRNAs make it possible to become a biomarker or therapeutic target. At present, cheRNA *PCA3* has been used in the clinical diagnosis of prostate cancer for its high expression, providing a reliable basis for the treatment of prostate cancer (Lee et al., 2011). Moreover, specifically targeting cheRNA *MALAT1* by antisense oligonucleotides (ASOs) can significantly reduce tumor volume, induce cell differentiation, and inhibit metastasis in a mouse mammary carcinoma model (Arun et al., 2016; Amodio et al., 2018). It is expected that more newly discovered cheRNAs will be useful in clinical diagnosis and tumor therapy with the development of advanced technologies.

However, challenges are obvious before cheRNAs can be applied in tumor targeted therapy on a large scale. Firstly, cheRNAs modulate chromatin homeostasis in many ways, but the mechanism of its binding to chromatin remains unclear. In addition to directly binding with protein and DNA, is there any other mechanism of cheRNA locating around chromatin? Secondly, RNA molecules have shortcomings, such as low transfection efficiency, off-target, and short half-life, which may greatly impact the drug delivery efficiency. Therefore, finding a more suitable drug delivery method is a priority. It has been proposed to use nanoparticles to improve the efficiency of targeted drug delivery (Wang et al., 2019a). In conclusion, clarifying cheRNA with its expression, structure, and mechanism is significant for the understanding of tumorigenesis, and it can also provide new insights for tumor diagnosis and targeting therapy.

## Author Contributions

JZ and TD designed and drafted the manuscript. HZ discussed, revised, and approved the manuscript. All authors contributed to the article and approved the submitted version.

### Conflict of Interest

The authors declare that the research was conducted in the absence of any commercial or financial relationships that could be construed as a potential conflict of interest.
